# Utilization of Natural History Information in Evidence based Herpetoculture: A Proposed Protocol and Case Study with *Hydrodynastes gigas* (False Water Cobra)

**DOI:** 10.3390/ani10112021

**Published:** 2020-11-03

**Authors:** Zachary J. Loughman

**Affiliations:** Zoo Science and Applied Conservation, Department of Biology, West Liberty University, West Liberty, WV 26074, USA; zloughman@westliberty.edu

**Keywords:** herpetoculture, evidence based husbandry, natural history, *Hydrodynastes gigas*

## Abstract

**Simple Summary:**

Many reptile and amphibian husbandry practices today are based on entrenched dogma, and not necessarily science. Folklore husbandry is animal care based on this dogma, where science does not drive the advancement of herpetoculture, and the adage, “this is the way we’ve always done it” does. Though not a novel concept, a resurgence in evidence-based husbandry approaches has infiltrated recent herpetoculture practice in direct response to folklore husbandry, challenging long standing practice. Herein, natural history information, in particular, diet, habitat, climate, behavior in the field, including spatial use and reproductive biology, serve as a primary source of information in evidence-based husbandry. With widespread availability through the internet, this information that once was difficult to attain is now accessible to the masses. A theoretical framework and protocol are presented that enable anyone who has internet access the ability to address key natural history questions directly allied to herpetoculture to determine thermal husbandry, food types and feeding frequencies, enclosure size and design, and breeding protocols for amphibians and reptiles in human care. A case study and assessment protocol for efficacy is provided for false water cobras to demonstrate this method.

**Abstract:**

Herpetocultural practices are based on norms driven by economy of space and time for keepers, with little scientific inference backing their practice. In recent years, a subset of herpetoculturalists have promoted evidence-based husbandry that relies on science and experimental design to generate husbandry practice. A theoretical framework and protocol are proposed herein that enables any individual who has access to the internet the ability to use various outlets of natural history information (scientific literature databases, social media sources, and weather websites) and previously published husbandry reports as evidence to drive the creation of novel herpetocultural practice. A case study is provided which compares readily available information on the care of *Hydrodynastes gigas* (false water cobra), such as online care sheets for the species, with the proposed evidence based herpetocultural protocol founded on natural history information and published care and captive breeding reports. Results were assessed for protocol efficacy and determined that the natural history informed evidence-based approach increased animal welfare and generated new information specific to the natural history of *H. gigas*.

## 1. Introduction

Herpetoculture, the maintenance and breeding of amphibians and reptiles in human care, has a long history in both zoological institutions and the private sector. Bateman’s [1] “The Vivarium” is considered by many to be among the first English-language publications providing guidelines for amphibian and reptile keeping, though several German publications predated Bateman [2]. The Viviarium and Bateman’s writings provided a foundation for what later would be deemed herpetoculture, once captive breeding became a focus. When discussing enclosure design, Bateman promoted naturalistic approaches specific to the species in human care. Interestingly, the practice of including nature in vivarium design, today known as naturalistic keeping, would fall out of favor in the future in certain herpetocultural circles. During Bateman’s time, herpetoculture was rarely practiced outside of zoological or academic institutions; that would change in the next 200 years. 

In the last 30 years of the 20th century, amphibians and reptiles gained in popularity as pets in the private sector. Care for these animals today varies from scientifically mediated approaches, to methods that lack any scientific backing. A review of 1980s and 1990s herpetocultural literature indicates that for many species a set husbandry norm was applied to entire taxonomic groups, becoming husbandry dogma. A spectrum from minimalist approaches of early zoos and select private hobbyists to naturalistic designs promoted by Bateman [1] and another group of private hobbyists existed in these guides. Diversity in temperatures, food types, enclosure types, and other husbandry attributes often were lacking.

Over the past century, increased consideration of animal welfare has led to awareness of animals needs in human care [3,4]. Much of these improvements have focused on mammals and birds, which are perceived as more intelligent than amphibians and reptiles [3,5]. Amphibians and reptiles only recently have received attention in this arena, demonstrating they too are capable of play behaviors [3,6,7], can be trained using operant conditioning [3], and need complexity of care equivalent to many mammals and birds to survive captivity [3,4,8,9]. This being said, minimalist keeping still permeates much of herpetoculture today, just in different forms [10,11,12]. The question is, is this minimalist approach justified?

Minimalist mindsets enable keepers in both the private and zoological sectors to maintain large collections in reduced spaces. Rack systems, where plastic tubs are used as enclosures and maintained in a drawer system with heating controlled via a thermostat appeared in the 1970s and 1980s [11], and are utilized for various reasons, including but not limited to rearing offspring, use as quarantine enclosures, and permanent housing for adult animals [11]. Private herpetoculture utilize racks that are associated with the husbandry of several species readily bred within the private sector, like *Eublepharis macularius* (Leopard Geckos) and *Python regius* (Royal/Ball Python), and several zoos utilize racks for off exhibit caging. Their use has proven controversial due to perceived welfare issues, specifically their limited space and the lack of illumination inside [10]. 

In the 1980s and 1990s following the increase in minimalist keeping a transition from keeping to breeding occurred, in the private sector especially [7,10]. “Care sheets” and in the last 10 years short care videos [13] have proliferated on the internet in response to amphibian and reptile popularity, and often serve as the primary source of information for most reptile and amphibian keepers and entry level herpetoculturalists. Though a species may have several to hundreds of care sheets/videos available, much of their suggested husbandry practice is monotypic and simplistic in nature. Information promoted on care sheets/videos normally encourages a more simplistic approach to keeping indicative of minimalist techniques [10]. Not all care sheets/videos lack detailed information; exceptions do exist. Published husbandry reports and articles specific to advancements in husbandry also exists for several species [11,14], though can be difficult to find, allowing care sheets/videos to dominate within the private sector as the source of information for most reptile and amphibian husbandry. 

Should information used to develop husbandry techniques be the result of careful scientific study of amphibians and reptiles while in human care? One could argue not necessarily, given anthropogenic environments are not where the species in question evolved, and therefore they should not set the precedence for husbandry. One could also argue husbandry attributes associated with herpetocultural practice should then be driven by observations and investigations in nature, where the species in question occurs naturally and evolved. Approaches similar to Case [15] and Tetzlaff [16], who used an evidence based approach comparing minimalistic with naturalistic care, and documented increased stress in *Terrapene carolina* (Eastern Box turtles) maintained in simple versus complex conditions, help support one approach over another. Regarding behavioral enrichment, reptiles time and time again have been shown in scientific studies to respond to complex captive environments [11,15,16,17], complex thermal repetoires [11,18,19], and complex diets [11,18] in ways that decrease stress and demonstrate choice. Evidence based husbandry has become a favorable herpetocultural approach [11,12,14], specifically to those who hope to avoid folklore husbandry practices [11,14]. 

Herein, a species’ natural history becomes the driver of herpetocultural practice, given at its core, natural history focuses on the who, what, when, where, and why of a species [20]. Natural history practice has also come under threat over the past three decades [20,21,22], and resultant of its short absence as a pillar of biology, natural history today is witnessing a resurgence [22]. It is important to note that defining what natural history is can be problematic, given that natural history practitioners range from field-based biologists, literary authors, to artists. Bartholomew’s [20] definition of natural history *sensu stricto* (see Section 2.1) is the definition most useful to herpetoculture, given its focus on the biological sciences, and the acquisition of field-based data on species to answer the previous questions. 

The world today is very different than Bateman’s [1], and via the internet, natural history information is readily available. Understanding what information is relevant, where to find this information, and how to organize information once gathered for herpetocultural practice can be overwhelming to individuals wanting to engage in evidence based herpetoculture. Once in hand, natural history data/information can be used to generate an evidence based herpetocultural protocol (EBHP) that can be employed for amphibians and reptiles in their care. Keepers tasked with designing exhibits and husbandry protocols with limited experiences, researchers looking for an approach to create enclosures and husbandry regimes for ex situ studies, and private hobbyists hoping to develop a method for natural history-based husbandry all could use a stylized protocol to approach evidence based herpetoculture. 

Animal husbandry, specifically herpetoculture has relied on natural history as a driver of husbandry practice; this approach is certainly not novel to this manuscript [4,11,12,14], though has been less relied on as minimalist husbandry approaches pervaded herpetoculture. What is novel herein is the development of a stylized approach and model on how natural history information can be collected and used to create an EBHP for amphibians and reptiles in human care from already published sources. Assessment of success is important, and methods are also presented and suggested for EBHP assessment, further driving the use of evidence over dogma. A case study is provided explaining this method for *Hydrodynastes gigas* (False Water Cobra) husbandry. *Hydrodynastes gigas* was chosen given the author’s extensive experience maintaining this species, and the trouble that existed early on when trying to determine what husbandry practices were best for *H. gigas*. Experienced herpetoculturalists are encouraged to challenge the model to increase its efficiency, or take this approach as a foundation and build on what is presented herein. 

## 2. Materials and Methods

### 2.1. Explanation and Development of Model

Natural history is a broad set of disciplines ranging from data driven observations of the natural world to literary discussions of human/nature interactions [20,22]. Within the context of natural history’s use in herpetoculture, natural history sensu strico (s.s.) as described by Bartholomew [20] provides evidence for herpetological husbandry. Bartholomew [14] proposed naturalists are in pursuit of very specific questions pertaining to their subjects of study. Answers to these questions are not ascertained from hypothesis testing, but rather gathered from observation and note taking in the field, and occasionally the laboratory. These questions are: (1.) What species is it? (2.) Where does the species live? (3.) How does the species survive? and (4.) How did the species end up here? When answered these questions provide useful information for herpetoculturalists. Table 1 explores each broad natural history question and provides more specific elements that contribute towards husbandry.

A fifth natural history question outside of Bartholomew’s [14] is presented in this list; (5.) What is the species’ life history? The study of life history has been a mainstay of organismal biology and a sub discipline of ecology since the middle of the last century. Given its focus on reproduction and maintenance of populations in nature, life history knowledge is intimately linked to captive reproduction, a principal objective of herpetoculture. Herpetoculture has specific husbandry questions it is trying to answer explicit to the welfare of amphibians and reptiles in human care. Those questions include: (1.) What is needed for the captive environment? (2.) What are the species’ physiological needs? (3.) What is needed to encourage natural behaviors? (4.) What are the species’ welfare needs? and (5.) What is needed for captive breeding? The answers to these questions should be data-driven and elucidate from environments where species naturally occur. Like the natural history questions, there are specific sub questions nested in each (Table 2), that when answered, could lead to effective husbandry protocols. 

**Figure 1:**
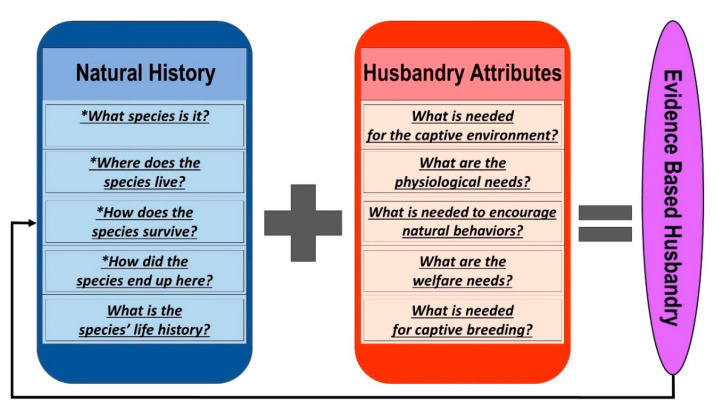
Model of the relationship of Bartholomew’s natural history questions and life history information along with questions specific to husbandry attributes when answered lead to evidence based husbandry, which in turn can lead to potential novel natural history observations (black arrow). * = Bartholomew’s questions.

One could argue evidence-based husbandry should rely on “evidence” that comes from natural history. In situ natural history data (habitat type, light levels, food types, etc.) then should drive specificities of husbandry (thermogradients, feeding frequency, temperature cycling, etc.), resulting in enclosure designs/husbandry practices that mimic natural environments where species occur and evolved. Specific questions are needed to drive the acquisition of facts/evidence, and Bartholomew’s questions serve this purpose nicely. Interestingly, opportunities arise if the species’ natural history is replicated in human care; novel natural history information can be observed and ascertained. So, the utilization of natural history to drive husbandry attributes leads to evidence based husbandry, which can result in the acquisition of unknown natural history information (Figure 1). 

### 2.2. Data Sources and Acquisition

Data are needed to answer the questions presented in Figure 1; where does it come from? More information is available today than ever via the internet, and it is here that information can be ascertained. Reliance on scientific literature and direct natural history observation is important to this practice, so understanding where these sources reside on the internet, how to gather this data, and what data to gather is paramount to this model of care. Utilization of free internet data sources thereby increases both its usability and utility. Google Scholar literature searches serve the basis for acquisition of species specific natural history information, and are particularly useful given returns not only include primary literature, but also relevant books that cite information associated with key phrases searched. Searches using a species scientific name can result in a single, tens, hundreds, or even thousands of results, not all of which are equally useful for EBHP development. Digital object identifiers (DOI) from publications can then be used to find article PDF’s that are returned in the Google Scholar search. 

An understanding of the different “types” of articles returned from these searches, and their husbandry importance is important for successful EBHP development. Books/monographs with “Amphibians and Reptiles of…”, where … would represent a specific region usually present species accounts for taxa occurring in the geographic region covered. Another key title phrase is “Field Guide to Amphibians and Reptiles of…”. Field guides usually focus on identifications/distributions at a regional scale and lack detailed natural history information. Sometimes, though, they are hybrids between monographic treatments and the typical field guide format, providing detailed biological information. Given the emphasis on where an amphibian or reptile is found, field guides can serve as information sources specific to the geographic distribution of a species being investigated.

Specific words/phrases in natural history/ecology journal article titles serve herpetoculturalists more than others, and lead to data presented in Table 1 ascertainment. In this instance, … would represent either the species of interest, or a closely related taxa that natural history information could be gleaned from in the absence of articles on the focal species. Searching for the following key title phrases, “The ecology of…”, “Behavioral ecology of…”, “Reproductive biology of…” “Diet of …”and “Feeding ecology/biology of…” can lead to the answers of several natural history questions the herpetoculturalist’s seeks (Table 3). These papers usually are resultant of multiyear efforts and often occur over the focal species entire activity season, providing insight into seasonal differences in ecology, behavior, and feeding. Additional title keywords that can lead to articles are terms focusing on species descriptions, species in an ecological modeling context, and the role species play in community structure. All of the latter’s focus on ecological and taxonomic questions begins to leave natural history’s umbrella, but include isolated data that can serve herpetoculture. For example, most species descriptions include species collections locales, and may include specific ecology and natural history sections as well. Life history trait-based analyses are useful for gathering natural history specifics such as diet type, life history strategy, and preferred habitat and can serve herpetoculture in food type and feeding frequency determination.

Another source of primary literature are brief natural history notes like those published in Herpetological Review and Herpetology Notes. These often focus on reproductive observations, habitat usage, size records and foraging behaviors and types, all of which are attributable to the development of an EBHP. Distribution records are a critical component of this protocol; a simple coordinate can lead to nearest city names to be searched in weather websites. Weather websites provide information specific to seasonal temperatures, average highs and lows, 24 h and monthly temperature differences, which can serve as the basis for thermal regimes for amphibians and reptiles in human care. Several printed natural history data sources (Table 3) as well as digital databases house this information. Specifically, museum collections, such as the Comparative Museum of Zoology at Harvard and large multi collection databases like VerTneT©, are readily searchable using scientific names, leading to distribution information (regions, counties, cities etc.) where the focal taxa has been collected.

Citizen science and social media sites also provide this information. Searching species scientific names in iNaturalist© can lead to site data, and, given each record presented in iNaturalist© was contributed by an individual, the opportunity presents itself to directly converse with individuals who observed the species in the field. Though limited in scope, isolates of behavior, macro/microhabitats utilization, and even prey species are garnered through searching these outlets, especially if in situ videos/pictures are provided. Social media, specifically Facebook© groups allied to regional amphibians and reptile assemblages, also allow individuals the opportunity to connect with people who have in situ experience with specific taxa. Utilization of information gathered from these groups should be cautious; being a member of a herpetology/herpetoculture social media group certainly does not make one an expert on a species. However, individuals who have extensive taxa experience frequently are members of these groups allowing networking opportunities. In the end, it is up to the individual gathering information for their EBHP to determine if individuals experience and advice is worth incorporation into their EBHP. 

In addition to reviewing natural history information, a review of species level herpetocultural literature for focal taxa and their close relatives should also be completed. Once ascertained, results from those works can be compared to the developed protocol for potential validation of approach. Furthermore, understanding what may not have or did work to breed or cause a species to succeed in human care previously can only benefit EBHP development. Herpetological Review has been publishing a herpetoculture section since the late 1970s, and several additional amphibian and reptile journals publish herpetoculture findings. This being said, most of these reports are only available as hard copies and are included in out of print periodicals like The Vivarium and The Herptile, as well as various Zoological Institution reports. Edited volumes exist specific to meeting proceedings, and several recent books including “The Complete” series by Eco Publishing focus on natural history driven herpetocultural approaches. Several amphibian and reptiles have undergone taxonomic revisions. When this occurs, prior genus or species names are abandoned in lieu of updated taxonomy. For the purposes of this protocol, it is important not only to search the literature for a taxon’s current name, but also those used prior to modern taxonomic adoption. Inclusion of literature written in languages outside the practitioners’ native language is also important. Several online translation tools exist, and so long as gathered articles can be uploaded into a translator, the language barrier can be overcome, and information can be included in protocol development.

### 2.3. Building the Husbandry Protocol from Data Gathered

Once gathered, data management becomes critical for protocol development. Utilization of spreadsheets with the questions headings in Table 2, one of which is provided (Table S1) are important tools for this step in the process. Emphasis on temperatures, food items, and macro-habitats used can be used to determine substrate, food items, feeding frequency, and the level of enclosure complexity needed. Enclosure type and design is directly determined from literature/distribution records, field observations, and other sources. For example, animals who require high humidity would need to be housed in enclosures that might differ structurally from those requiring xeric conditions. 

Lighting needs also can be determined by correlating habitat types to associated UVB levels. Amphibians/reptiles that are diurnal and active in open habitats likely correlate to UVB levels above 5.0 on the Fergusson Scale [23]; those from forested environs could require lower levels. This information can be deduced from distribution accounts, “Ecology of…” manuscripts, as well as other sources. Information specific to feeding frequency and growth rates are allied with “Ecology of…”, “Diet of…” and “Feeding biology of…” manuscripts, which often include food type and feeding frequency seasonality information. “Behavioral biology of…” manuscripts prove useful for answering all sub-questions included in question #3 in Table 2, as well as other questions. Often these papers focus on behaviors allied with specific demographics, habitats, seasons, and times of day, which can drive husbandry essentials such as number and frequency of hides, space requirements, thermal gradients, and the need for seasonal cycling. Given amphibian and reptile’s reliance to climate and season for their ontogeny, reproduction, and basic wellbeing, this information is important, especially for answering Table 2 questions 1a, 3c–e, and 5a. 

Locations gathered from the aforementioned sources determine the climate and seasonality allied with amphibian/reptiles in need of EBHP development. Specifically, the following order of operations can lead to development of a thermal regime in human care:

Location determined → Location found in Google Maps → Location searched in weather website

Weatherspark.com© is useful to determine the climate an amphibian/reptile occurs in. This website uses weather data to create figures that quickly allow herpetoculturalists access to seasonality, thermal regimes, maximum and minimum temperatures, seasonal humidity, and precipitation amounts where a species in question lives. What is missing from this equation is microclimatic variables, most of which can only be determined directly from field observations or literature reviews.

The final, and arguably most important, source of natural history information is in situ investigation/observations by herpetoculturalists themselves. All of Table 2’s questions can be answered through field studies and observations. While every opportunity should be undertaken for keepers to visit the natural habitat of their charges, logistical and financial limitations may keep that from happening. Time in the field offers opportunities to validate husbandry attributes, enabling individual variable fine-tuning. Table 3 presents sources of information and the specific natural history questions those sources can be used to answer.

### 2.4. Determining if the Protocol is Successful at Increasing Welfare

Once EBHP development has occurred and natural history evidence ascertained, EBHPs can be executed. Now what is needed are metrics to determine what is, and what is not contributing to animals’ success under human care. Benn et al. [24] reviewed several welfare protocols used for mammals and determined that their application was possible with reptiles if class specific changes were willing to be employed, specific to welfare determination metrics. To that end, various metrics can be used to determine success, all of which should focus on information generated by the animals themselves. Cues/metrics include but are not limited to neonate/juvenile growth, stable weights in adults, behavioral responses to their environment, and hormone levels. Anorexia is a standby, nonintrusive measure of stress. Several amphibian and reptile species undergo seasonal anorexia, so an understanding of a taxa’s natural history is important if appetite loss is indicative of increased stress or seasonality. Prior to being introduced to the new husbandry regime, animals should be weighed to determine baseline information; weight loss could be interpreted as a stress response, but careful understanding of the species natural history is important at this step [25]. 

Understanding natural behaviors versus stress behaviors can also be used as a welfare tool. Lack of activity, specifically typical thermoregulatory, stylized inter-individual, and lack of foraging and hydration behaviors all can be used to asses’ welfare success. Understanding what constitute “normal” behavior is both a byproduct of research and experiences with the species in question. Increased stereotypies, such as constant interactions with transparent boundaries (ITB; [25]), nose rubbing, and perpetual pacing all could indicate the EBHP is causing stress. Remedying these issues is reliant on determining which aspects of Table 2 are stress inducing. Individuals with access to laboratories, especially those at zoological institutions and universities, could use corticosterone levels as direct measures of stress response, though use of this metric has yet to be perfected for herps [26]. Determination of corticosterone levels prior to initiation of husbandry protocol, during acclimation, and post acclimation allows verification of stress responses. Breeding seasons, brumation, and periods of extreme temperatures all can fluctuate corticosterone levels, making baseline determination for different time points over the year important prior to EBHP implementation [26]. In addition to corticosterone testing, understanding behaviors that are indicative of low/high stress response allows for quick determination of protocol success. 

### 2.5. Husbandry Informing Natural History

When data acquisition and key husbandry questions have been answered, natural history and husbandry data gaps can be identified. It is now that the full utility of this approach becomes apparent. For instance, if following a search zero reproductive biology information is found on a species that then reproduces in human care, a data gap has been filled, and this information should be published as a natural history note or as a primary publication. In this regard, the protocol is fueling natural history information, giving back to what developed it. 

### 2.6. Study Species and Initial Care Methods

To demonstrate this approach a case study is presented utilizing the previously described methods with an EBHP devised from natural history information and previous husbandry and breeding reports for *Hydrodynastes gigas* (false water cobras), a large dipsadid colubroid native to Argentina, Bolivia, Brazil, and Paraguay [27]. Two female neonates, one juvenile male, and two adult (1♂:1♀) snakes were aquired in the winter of 2017. Two additional animals were produced from a clutch of eggs the initial pair of adult animals produced in the summer of 2018. At the onset of care in 2017, the original two neonates were maintained in 10 g aquaria, and the subsequent juvenile male, and adult male and female were maintained in 40-gallon aquaria. Daily temperature gradients of 24–35 °C were employed and UVB lights were not used. Mulch substrates were used, and cage furniture consisted of a single hide. Snakes had access to a water bowl. Zero behavioral enrichment was incorporated, and animals were fed a single pre-killed laboratory rat and mouse once a week. This husbandry protocol was developed from books [28] and snake husbandry websites. Neonates incorporated into the collection in 2018 husbandry followed the developed EBHP described in the results section.

## 3. Results

### 3.1. Initial Internet Search Results for Care

Four dedicated articles existed specific to *H. gigas* husbandry in human care prior to EBHP and this species was briefly discussed in several care protocols for colubroids. All four articles were from European herpetocultural periodicals, were not available electronically, though two [29,30] were attained and ultimately incorporated into the EBHP. Melani [29] was obtained after initial EBHP development, and verified several husbandry attributes integrated into the *H. gigas* EBHP. Specifically, Melani [29] was the only reference found that encouraged seasonal cycling prior to breeding, cessation of feeding during winter months (see below), and noted double clutching in *H. gigas*. A Google search for “False Water Cobra care sheet” and “False Water Cobra care” generated 10 pages of results. Most care sheets recommended 4’ Length × 2’ Height × 2’ Width (1.2 m × 0.7 m × 0.7 m) enclosures; no care sheets recommended racks as *H. gigas* enclosures, and several sheets explicitly spoke out against racks. Suggested ambient temperatures ranged from as low as 20 °C to as high as 35 °C, with an average of 27 °C. Diet suggestions ranged from simplistic laboratory rodent only diets, to more diverse diets including frog legs, chicks, quail, and fish. No care sheets recommended seasonal cycling, and all breeding protocols except Melani [29] simply stated snakes should be put together when eggs were desired. Incubation temperature was consistent across care sheets at 28 °C. 

A Google Scholar search for “*Hydrodynastes gigas*” resulted in >25 pages of journal articles, published abstracts, book chapters, monographs, and book titles. Of these, 21 journal articles and 2 monographs were downloaded and used to generate an EBHP. Two publications in particular proved extremely valuable for natural history inference. The first, Giraudo et al. [31] covered most Table 1 natural history questions, though proved problematic since it was written in Spanish. The entirety of the article was copied and pasted into a Word document, then translated with Google translate to English. Another publication, Lopez and Giraudo [32] focused on *H. gigas* feeding ecology, and coupled with Giraudo et al [31], provided a wealth of information on food items, feeding frequency, macro and microhabitat utilization, diel behavior, seasonal behavior, and activity levels. These two manuscripts contributed much of the evidence used to develop the EBHP for *H. gigas*. Additionally, several articles focused on distribution [27,32,33,34,35,36].

*Hydrodynastes gigas* has a large range that covers most of northern and central South America east of the Andes Mountains, so determination of which portion of *H. gigas* distribution to base husbandry climatic variables on was an issue. Conversations on the Facebook groups False Water Cobras and The False Water Cobra with keepers and breeders who had extensive experience with *H. gigas* led to the deduction that most *H. gigas* maintained in captivity in the United States were likely imported from Paraguay or Bolivia. Further conversations determined more animals likely occurred in the U.S.A. from Paraguay than Bolivia, and Campbell and Murphy [37] reported on the reproductive biology of animals collected from the vicinity of Asuncion, Paraguay, so distributional records specific to Paraguay drove searches for husbandry climatic variables more so than other distribution records. 

A search of the Harvard Museum of Comparative Zoology’s MCZ_BASE_ database for *Hydrodynastes* returned five *H. gigas* records, two of which had decimal degrees from Paraguay, which were searched in Google Maps. One record came from the Argentina/Paraguay border 10 kilometers west of Asuncion, Paraguay, and coupled with Campbell and Murphy [37] reporting on specimens collected from this region, Asuncion climate was used for husbandry protocol development. A search of Asuncion, Paraguay from the website Weatherspark.com determined that the “summer” season occurs November through March, with high temperatures around 33 °C and lows around 22 °C, and “winter” season occurs May through July, with temperatures ranging from highs of 25–23 °C and lows of 16–11 °C. Precipitation ranges from 20% to 40% daily probability during summer, and 15–20% during winter months. Photoperiod does fluctuate, with 12 h L/12 h D of light September through March (6 months), 11hL/13hD during transitional months, and 10 h L/14 h D during the 2 coldest months of the year. Humidity levels differ seasonally with summer months having up to 85% humidity and winter months dropping to as low as 6%. Photoperiod indicated that during summer months, solar radiation is high (5.5–7 kWh) and drops moderately in winter (3.4–4.2 kWh). This information was used as the basis of captive photoperiod, UVB usage, thermal regimes, and temperature/cycles for *H. gigas* (Table 4).

### 3.2. What is Needed for the Captive Environment?

Much of the literature indicated that *H. gigas* is active [27,31,32] and spends ample time hunting during daylight [25,26,27]. Knowing this, 1.8 mL × 1.0 mW × 1.0 mT PVC enclosures were procured to house adult snakes. Enclosures size was chosen because their dimensions were the largest that could be placed in the space ascertained for *H. gigas;* larger enclosures would have been chosen if space was available. PVC enclosures were chosen because of their lightweight and durability, and not because of any natural history information. Substrate composed of a 10–15 cm layer of cypress mulch mixed with wood pellets, leaf litter, and sphagnum because it both absorbs fecal waste for spot cleaning and maintains appropriate humidity levels. This depth was chosen because *H. gigas* were reported to occasionally burrow in loose substrates [25]. Weatherspark.com also indicated marked seasons occur in Asuncion, so maintenance of a thermal regime for the majority of the year with a high temperature of 31 °C, night time low of 22 °C, and a daily ambient of 25 °C was utilized for 10 months of the year excluding winter. Light levels differed seasonally at Asuncion, so a light (L) dark (D) cycle of 13 h L/11 h D in summer and 10 h L/14 h D in winter was employed.

All retrieved habitat references indicated *H. gigas* prefers open wetlands and scrub forest, and rarely was encountered in primary forests or jungles [27,31,32,38]. Most *H. gigas* observations occurred midmorning, though several observations occurred mid-day, and iNaturalist records (n = 50; 15 June 2020) indicated *H. gigas* was frequently observed late morning and afternoon hours. These results were indicate *H. gigas* could benefit from bright light and UVB exposure. LED lights were used to illuminate enclosures and, when possible, 5.0 UVB bulbs were employed. Interestingly, when provided UVB *H. gigas* chose to bask under UVB bulbs more so than heat lamps providing the same temperatures. Multiple hides were utilized; a few social media posts from the Facebook group “Fotografiando la HERPETOFAUNA Argentina” indicated that *H. gigas* are occasionally found hiding under human debris, in tall vegetation, and under logs, leading to inclusion of these items in *H. gigas* enclosures.

### 3.3. What are the Physiological Needs?

Based on natural history notes specific to feeding [27,34,35], a snake community analysis investigating diet breadth in snakes including *H. gigas* [38], Lopez and Giraudo [32] and Giraudo et al. [31], it was determined *H. gigas* should have as broad a diet in human care as possible. Most studies indicated amphibians [26,27,33], fish [25,26,27,33] and reptiles [26,27,33] make up the bulk of *H. gigas* diet in the wild, and birds [26,27,33] and mammals [26,33] are taken sparingly. All ecological work noted *H. gigas* strong affinity to aquatic and wetland habitats [25,26,27,28,29,30,31,32,33]. Resultant of this, water basins were provided large enough for them to submerge their entire bodies. 

The development of a feeding regime for *H. gigas* included laboratory mice and rats and chicks based on Giraudo et al. [31]. Multiple mouse/rat demographics were fed to provide variability in protein, fat, and sugars. Frog legs and trout were also included since both were taxonomically relevant to observations made in nature [26,27,33]. Diversity was key; this being said the bulk of *H. gigas* diet was still rodents. Multiple small food items twice a week for all demographics was the feeding frequency developed. This determination came straight from Lopez and Giraudo [32] and Giraudo et al [31], who noted many snakes had food items in their gastrointestinal tracts, leading them to deduce *H. gigas* feed frequently and whenever the opportunity presented itself. Further observation in captivity determined most prey items are voided within 72 h of consumption, further validating this feeding regime. 

### 3.4. What is Needed to Encourage Natural Behaviors?

As stated previously, most encounters with *H. gigas* occur when snakes are patrolling habitats. Given the frequency of these observations, as much space as possible were utilized in EBHP development. Hides were also needed, though placement was important to enable opportunity for privacy across a broad thermal regime. For this reason, two hides were utilized per enclosure, one in the “hot” and the other in “cooler” portions of the enclosure. *Hydrodynastes gigas* were observed basking in several iNaturalist posts, so a thermal gradient was provided to enable basking behaviors and a seasonal cycle was created. UVB was also employed to promote natural behaviors [31]. Seasons were converted to follow the northern hemisphere, with the summer season beginning in February and lasting through mid-October; winter season occured mid-November through mid-January. Temperatures for those seasons (Table 4) were deduced from annual weather data for Asuncion, Paraguay.

### 3.5. What are the Welfare Needs and Concerns?

*Hydrodynastes gigas* spends considerable time roaming and patrolling [31,32,33,37,38] so roaming opportunities were granted. All animals were given opportunities once every three days to roam a 3m x 8m room with hiding places and climbing opportunities for several hours. Food items also were placed randomly throughout the room to promote encounter behaviors similar to what would occur in nature [27,31,32,33,37]. Welfare assessment was completed through use of corticosterone level determination from feces, as well as direct observation of *H. gigas* behavior. An ethogram was developed to determine which behaviors occurred frequently and represented “normal” behaviors. The list of behaviors was deduced from direct observation in human care and the completed literature review of *H. gigas*. From this, an abnormal behavior was noticed where snakes would pace along the front glass of their enclosure. When this was observed, efforts were put forth to allow individuals to roam, and in all instances, the behavior extinguished within a matter of days. 

A potential welfare concern was the care of neonates, as this demographic lacked natural history information, representing a natural history information data gap. Giraudo et al. [31] noted that *H. gigas* are fecund, and produce large clutches relative to other colubroids. The author theorized this was an evolutionary response to predation on neonates, given the large suite of predators neonates need to avoid in the wetlands where they occur. Providing neonates both privacy and security was an aspect of neonate *H. gigas* welfare. Rack systems with mulch substrates and hides were used for neonate care, specifically due to the privacy they provide. These systems were only used until animals were feeding readily and had taken 10 meals, at which point they were moved from rack systems to open enclosures, where light levels and thermal parameters previously mentioned could be employed. 

### 3.6. What are the Captive Breeding Requirements?

Three sources returned from Google Scholar proved useful for development of a reproductive plan for *H. gigas*. Campbell and Murphy [37] reported that females (*n* = 3) collected from the vicinity of Asuncion, Paraguay laid 6, 8, and 11 eggs for an average of 8.3 eggs/clutch. Campbell and Murphy cited Vogel [39] who reported 14, 29, 36, and 42 eggs/clutch from a population in Brazil for an average of 30.2 eggs/clutch. Furthermore, Campbell and Murphy noted that eggs were laid in late spring/early summer. Giraudo et al. [31] investigated follicular development and determined that both ovarian and vitellogenic follicles were present in snakes all year. Oviductal eggs began to occur in late spring and were present through mid-summer, with the majority of egg laying occurring early to midsummer. Clutch size ranged from 11–36, with an average of 23 eggs/clutch. Male testicular volume was stable for all months, though rolled and opaque vas deferens (non-active) were present in individuals during summer months. Food was found in the digestive tract of females with oviductal eggs, indicating *H*. *gigas* likely feed throughout egg development, or just prior to parturition. Finally, Bels [40] described courtship behaviors in *H. gigas*, indicating males do not bite females, instead aggressively pursue them. Courtship lasts for several hours, and normally occurred in the morning.

The previously mentioned seasonal cycle was developed where animals were fed and prepared for breeding during summer months. Following winter brumation, temperatures were increased and at this time males were placed with females for 48h periods of time, removed from females for 48h and this cycle repeated until either the male showed no interest, or 5 visual confirmed copulations were observed. Once either of the previous were met, males were removed, and females were fed on a normal feeding regime during follicular development and vitellogenesis. Females were offered food after pre-lay sheds until they showed no interest. Following oviposition, females were fed normally to regain fitness and not placed with males again until the following late winter/early spring when this cycle was repeated. Eggs, when laid, were incubated at 28 °C based on Campbell and Murphy [32], and all neonates following hatching were weighed, measured, and compared to Campbell and Murphy’s [32] results. Neonates were fed small food items two to three times a week. Steady growth was expected following observations made by Giraudo et al. [31]. 

### 3.7. Assessment of Protocol Success

The EBHP developed for *H*. *gigas* was assessed for effectiveness based on evidence of stress, growth, and reproductive success. The protocol was developed in the winter of 2017 and adapted consistently from that time through present (June 2020), during the duration of which records were kept specific to feeding, growth, reproductive success, and behavioral observations. Individual feeding rates were consistent through the spring and summer seasons of 2017–2020, and reduced during the winter season to little or no activity; this is consistent with observations made by Giraudo et al. [26] and Lopez and Giraudo [31] in naure. Body condition in all animals was indicative of normal condition utilizing the previously mentioned feeding regime and has remained consistent. Length to mass ratios for animals in care equilibrate to those recorded from nature [25]. 

Growth rates for all neonates and juveniles were reflective of field measurements for similar demographics [26], with the exception of the original two females which displayed rapid growth over the first 12 months of life. Specifically, both females increased in mass from 61 g to 2.0 kg (3.2 K% increase) and 53 g to 2.1 kg (3.9 K% increase) in one year. Growth was above average based on natural history information [31]. The original females were not winter cycled as juveniles, and fed summer month rates yearlong. Subsequently, when the two neonates born in 2018 were added to the collection, they were overwintered with the same feeding schedule as adults and displayed mass/snout vent lengths (SVL) similar to demographics reported in Giraudo [31]. This is now the adopted strategy for rearing *H. gigas* by the author. 

Enrichment and neonate care were also assessed. Online sources state *H. gigas* perceived reliance on water for soaking is in response to animals being too warm, and water basins may not be necessary for *H. gigas* care. *Hydrodynastes gigas* utilized water basins frequently, and water was utilized by all individuals during winter months. *Hydrodynastes gigas* skin temperatures were recorded with an infrared temperature gun when observed basking, and temperatures ranged from as low as 20 °C to as high as 31 °C. Animals’ temperatures in water basins ranged from 18–27 °C. Based on these observations, it was hypothesized that water basins were important for *H. gigas* welfare, given their frequent utilization during all seasons. 

Neonates maintained in racks usually began feeding within two weeks of hatching after post-natal sheds and were transitioned to enclosures within two months of birth. Less than 15% of neonates/juveniles displayed stress behaviors following this transition, specifically anorexia and IHB. To increase welfare, individuals displaying the latter were returned to racks and slowly transitioned to enclosures. Rack drawers were left open for brief periods of time enabling neonate’s observation of the room’s daily activities. Usually, 3–5 of these sessions were required to successfully transfer neonates to enclosures. To date, all individuals maintained by the author (*n* = 14) have been transitioned to PVC enclosures. Growth and feeding rates were used to validate this strategy for neonate and juvenile care, and efforts are underway currently to include corticosterone level determination specific to neonates as well. 

Behaviorally, if *H. gigas* are stressed they create a hood with the cervical region of their bodies. This hooding behavior was used to assess enrichment opportunities and their overall effectiveness. Animals were regularly allowed to explore the room holding their enclosures; roaming behaviors were encouraged. Prior to adoption, all animals would frequently hood during interaction periods. Following inclusion of roaming behaviors, all animals have reduced hooding to situations when they are startled; this measurement is subjective in nature and an area of future development of assessment within the proposed EBHP. Beginning in winter of 2020, a more objective measure of stress was adopted utilizing corticosterone levels in all aforementioned snakes. Levels were indicative of animals that were not stressed (range 14.0–20.0 ng/g) [26], and indicate that the EBHP at the time of testing was not inducing stress in the snakes.

Both original neonate females reached sexual maturity in the fall of 2019 consistent with Giraudo et al. [26] and cycled for breeding during the winter/spring of 2020. Both females produced clutches, with the larger female producing 23 eggs and the smaller female producing 24 eggs; 100% of eggs were fertile from both clutches. Females were fed according to the diet protocol, and both subsequently produced second clutches without access to males between clutches. The larger female produced 24 fertile eggs and five nonfertile eggs, and the second female produced 15 infertile and one fertile egg; the latter female likely would have produced a viable clutch had she been placed with a male. Double clutching was not reported in any of the natural history data the author had access to for initial protocol development (Author in prep.). Melani [29], a herpetoculturalist, was the first to document the phenomena. 

Every effort was undertaken to incorporate in situ elements determined from natural history information or species-specific husbandry reports to drive all husbandry decisions. Some husbandry decisions were made based on convenience (enclosure size) that were not based on natural history observations; in these instances, husbandry augmentation occurred to improve welfare and incorporate natural history observations (free roaming of rooms). Observations specific to growth, reproductive success, consistent feeding, and corticosterone levels were utilized to determine that the EBHP developed for *H. gigas* from spring of 2017 to spring of 2020 was conducive for their propagation, and increased their welfare compared to the original husbandry strategy employed by the author in the winter of 2017.

## 4. Discussion 

Natural history information and previous husbandry reports provided a basis for the development of an EBHP for *H. gigas* husbandry. Much of the protocol developed differed from husbandry data provided by a generic search of the internet. Online suggestions of thermal husbandry and light/dark cycles differed from natural history sources, and appeared based more on herpetocultural dogma, and not on science. Furthermore, enclosure size suggestions from most internet sources were smaller in volume than those deduced using an evidence-based approach. In actuality, the chosen enclosure size was an artifact of space availability, and should be as large as possible for these active snakes based on ascertained evidence. Zero websites reported on enrichment needs and possibilities for *H. gigas*. Given the diminishment of stress related behaviors (hooding, mock striking, etc.) following inclusion of regular enrichment opportunities, it is hypothesized that enrichment likely had a positive effect on the welfare of this species. Similarities between available information online and the evidence-based protocol did exist in incubation temperatures, diet, and the denial of rack systems as appropriate long-term housing for juvenile and adult *H. gigas* in human care, though they were used in the current study for neonates for the first 1–2 months of life. Zero generic search items returned suggested means of assessing efficacy of care, which is the primary driver of evidence-based husbandry.

The goal of this publication was to propose a model that anyone can employ through use of the internet and previously acquired field data, enabling herpetoculturalists the ability to utilize natural history and published care and breeding accounts to drive evidence-based husbandry. Natural history-based approaches to herpetoculture are in direct opposition to Folklore Husbandry, given their reliance on the acquisition of husbandry methods from natural history sources in lieu of herpetocultural dogma. Folklore Husbandry, a product of this dogma, is pervasive throughout herpetoculture, and through use of this evidence based approach, keepers are put in the position to question everything and rely on science and natural history to conclude husbandry practice over word of mouth communication. Herpetoculture today is beginning to be based on sound science, though traditional beliefs still permeate the discipline deeply [10,11]. Thermal husbandry is an example where science, and technology converge. Today, many herpetoculturalists heat their reptiles with Mercury Vapor bulbs that also provide UVB radiation. Only two decades ago, rarely anyone utilized Mercury Vapor bulbs, and instead many reptiles were being heated by “hot rocks”. UVB was rarely employed.

UVB still is a controversial subject amongst herpetoculturalists, especially in snake husbandry. Snakes lack pineal glands which lizards possess, and are thought to contribute and drive basking behaviors needed to ensure enough UVB radiation has occurred for Vitamin D_3_ synthesis and calcium uptake [41,42,43,44]. Herpetologists deduced that snakes likely do not require UVB radiation in D_3_ synthesis decades ago, and this has since become herpetoculture dogma. Recent experimentation with UVB exposure in snakes challenges this determination, and has consistently demonstrated elevated vitamin D_3_ levels in blood plasma and higher calcium deposition in bones after UVB exposure [37,39,40]. A single study conducted on *P. regius*, a mainstay of private herpetoculture, determined that no significant differences in vitamin D_3_ plasma levels occurred in response to UVB exposure [42], which many in herpetoculture interpret to mean UVB is not necessary or beneficial for *P. regius* or snake welfare. 

What is interesting is the number of herpetoculturalists who gravitated to this single article [42] and readily cite it to support not supplying UVB to their charges, and fail to acknowledge the other three articles on snakes that demonstrate a “ positive”physiological response to UVB. Natural history driven evidence-based approaches could add inference to this issue. Could it be that the natural history of a snake, and its subsequent evolution to meet the demands of the ecological theater it occurs in might drive the importance of UVB radiation in its physiology? Maybe UVB level needs differ between snakes in different habitats? A viper resting on a jungle floor might require different UVB levels than *H. gigas* patrolling an open, wetland habitat. 

Results from this study demonstrated a behavioral shift towards UVB radiation in *H. gigas*. An experiment was performed where UVB bulbs were moved to different locations, after timers turned off at night. *Hydrodynastes gigas* often would be observed “basking” in tight coils under these bulbs the following morning, especially gravid females. Equal amounts of heat were provided by additional bulbs, providing evidence that UVB could provide some kind of behavioral attraction. Though anecdotal, evidence was produced through observation of these snake’s behavior indicating *H. gigas* at least gravitate towards UVB when provided, hence its continued inclusion in the developed protocol. Utilizing an evidence-based husbandry framework in tandem with a UVB exposure experiment, inference into this pressing herpetocultural question might be garnered in a way that evolutionary history could help explain. This is the advantage of this approach to herpetoculture. If utilized, it will lead to questions, which likely will lead to herpetocultural advancements. 

Folklore husbandry occurs in zoological institutions as well as the private sector. Mendyk [11] addressed folklore husbandry in zoo herpetology sections, noting that when asked why thermal, hydric, and physical husbandry techniques were employed, most keepers could not cite the science driving these practices. Furthermore, Mendyk [10] noted that enclosure space often goes unused, and a status quo approach of substrate, stick, water, hide leads to stagnation in natural behaviors and the genesis of stereotypies and hypobehavior. Inclusion of the proposed natural history-based model would lead keepers charged with its employment the knowledge of “why” they are employing a given substrate, light, or piece of cage furniture.

Even with the focus on natural history, it is folly to think that the complete ecology of an amphibian or reptile can be replicated within the confines of an enclosure. Space limitations, incomplete matching of thermal needs, lack of access to natural seasonal cues, and equal consumption of food to that observed in nature by animals with reduced home ranges could lead to negative outcomes even with the best intentions and use of this protocol. Natural history information should be used to develop a list of items in a captive diet, but the quantity and feeding frequency of those items may need to be altered due to possible lack of activity or shift in behavior in human care. To rectify issues of this nature, it is important for keepers to observe their animals frequently, and if needed make subtle adjustments in care to offset potential negative health effects, like the potential for obesity in response to lack of activity.

Enrichment strategies should be used to promote natural behaviors needed to maintain adequate body condition and mental stimulation. To this end, it is important for keepers to use the information found in protocol development in conjecture with space to maximize its use. In the case of *H. gigas*, a natural history based enrichment protocol was developed where snakes were allowed to free roam a complex space for several hours once every three days. Ideally snakes would have access to this space daily; logistical issues prevent this from happening. This being said, when allowed to roam most animals were in perpetual motion for the duration of their room access, which mirrors several sources [26,27] observation that *H. gigas* are active snakes. Roaming grants snakes the opportunity to burn calories, and engages them mentally, and was used to extinguish a sterotypie that other *H. gigas* keepers had difficulty extinguishing [45]. The decision to allow animals this activity was derived entirely from the observations of biologists in the field [31], and is the tie in back to natural history information.

Another important driver of this approach is assessment of husbandry practice. Practitioners are encouraged to complete literature searches, search social media and citizen science outlets, and check the weather of locations used to generate their EBHP frequently. Michaels et al [14] also promoted the use of natural history information to drive evidence based husbandry practices for amphibians and noted that a hindrance to evidence based husbandry was the lack of natural history information for most amphibian taxa. Many reptiles lack detailed “Ecology of…” and other natural history studies as well. Mainstays in herpetoculture like *Pantherophis guttatus* (Corn Snakes), *P. regius* (Royal Python), *E. macularius* (Leopard Gecko) and *Correlophus ciliatus* (Crested Gecko) lacked dedicated “Ecology of…”, “Diet of…”, “Reproductive biology of…” manuscripts as of June 2020. All of the aforementioned species did have isolated natural history notes, as well as journal articles written about them, just not with the emphases which proves so useful to this protocol.

While dedicated natural history publications may be lacking for many species, this approach’s reliance on distribution information to ascertain seasonal and thermal husbandry attributes at the very least provides a baseline of care. Detailed information on microhabitats and their thermal properties cannot be deduced from broad weather websites and was pointed out by Michaels [12] as a need for effective evidence based herpetoculture. Interestingly, if seasonality is matched for a species that lacks natural history information, opportunities present themselves for herpetoculturalists to generate facts that aid the science of herpetology. Through creating multiple microhabitats and trying to replicate thermal dichotomies in enclosures, herpetoculturalists could fill voids in knowledge by noting preferences and behaviors associated with given temperatures ex situ. Conservation of species is dependent on a solid understanding of natural history [11,20,21,22], and this avenue of herpetoculture has potential to aid the conservation process. The union of herpetoculture, herpetology, and conservation biology is possible through use of evidence-based herpetoculture. 

The adage “Chance favors the prepared mind” is applicable here, and when novel natural history observations are made in the field or behavioral observations are made in the captive environment that prove useful in understanding a species biology, every effort should be made to publish those observations in herpetological and natural history based journals or similar outlets. This in turn feeds the pot of knowledge herpetoculturalists are dependent on in developing their husbandry practice. While academic and zoological professionals are encouraged to do this, herpetoculturalists in the private sector to should engage in this practice, and reaching out to professional biologists for assistance should be encouraged; observations that are made in the private sector can have just as much significance as those made in the laboratory, zoological institution, and field by professional herpetologists. 

Another advantage of this approach is keepers become engrossed in the natural history, biology, and conservation of their charges. Too often animals are maintained in human care for superficial reasons, and keepers often lack an understanding of the basic biology of their animals. When natural history information is ascertained and an EBHP developed following the methods proposed herein, keepers are exposed to the evolutionary history, ecological complexities, and natural history of amphibians and reptiles, which will/should lead to a deeper fascination and appreciation of amphibians and reptiles in their care. As Baba Dioum so eloquently stated, “In the end we will conserve what we love, we will love only what we understand”. This approach to herpetoculture leads to this conclusion, and that alone justifies its practice.

## 5. Conclusions

Evidence based husbandry strategies pushes the field of herpetoculture forward, and combats Folklore Husbandry by basing herpetocultural practice on science and not dogma. Evidence for husbandry attributes should be based on natural history observations of amphibians and reptiles in situ, given natural history is resultant of evolution, and therefore intimately linked to the survival of taxa. Utilization of evidence based husbandry protocols founded on natural history information utilizing the protocol proposed herein can lead to more efficient husbandry, increased animal welfare for amphibians and reptiles, increased keeper knowledge, and leads to the genesis of new natural history information, which can improve herpetocultural practice and add new knowledge of a taxa’s ecology, biology, and natural history.

## Figures and Tables

**Table 1:** Bartholomew’s proposed natural history questions and their proposed relationship to herpetoculture. Bolded questions are Bartholomew’s broad questions (numbers) with sub-questions (letters) that fall under them. Question #5 which was added by the author and is not a Bartholomew question.

**1. What species is it?**
a. Species description.
b. Systematic revision.
c. Evolutionary analysis.
**2. Where does the species live?**
a. Global distribution.
b. What biomes does it occur in?
c. What broad climatic patterns impact species?
d. What seasons exist where species occurs?
e. What macro/micro habitats exist where species occurs?
**3. How does the species survive?**
a. Thermoregulation and thermal needs?
b. Forage types and frequency?
c. Water needs?
d. Specific shelter needs?
e. Specific causes of stress?
f. Diel/circadian rhythms?
**4. How did the species end up here?**
a. Drivers of inter/intra specific competition?
b. Behaviors and behavioral ecology?
c. Species functional and realized niche in nature?
**5. What is the species’ life history?**
a. What is the species’ age at sexual maturity?
b. What is the species’ life span?
c. What is the species’ fecundity?
d. What is the species’ parity?
e. Do ontogenetic shifts occur in this species ecology?
f. What is the species’ life history strategy; r or K?

**Table 2:** Herpetocultural husbandry questions/attributes needed to ensure amphibians and reptiles exist/survive in human care, and the associated Bartholomew natural history questions that lead to husbandry inference.

Husbandry Question/Attribute	Bartholomew Natural History?
**1. What is needed for captive environment?**	
a. Enclosure type	1–3
b. Enclosure dimensions (L-W-H for adult)	2–3
b. Substrate type	2–4
c. Thermal needs—maintenance	2–4
d. Enclosure furniture type/amount	2, 5
e. Light needs	1–3
e. Light cycle needs	1–3
f. Relative humidity needs	1–3
**2. What are the physiological needs?**	
a. Food items	3
b. Feeding frequency	3
c. Requirements for "normal" growth	2–3, 5
**3. What is needed to encourage natural behaviors?**	
a. Space requirements	2
b. Hide availability	2–3
c. Thermal gradient	2–3, 5
d. Seasonal cycling—”Summer” cycle	2, 5
e. Seasonal cycling—“Winter” cycle	
**4. What are the welfare needs and concerns?**	
a. Space requirements	2–5
b. Privacy requirements	2–3, 5
c. Methods for welfare assessment	
d. Enrichment possibilities	
**5. Captive breeding requirements**	
a. Seasonal cycling needed?	2–3, 5
b. Incubation temperatures?	5
c. Feeding regime pre-mating	2–3, 5
d. Care of neonates—ontogeny	3, 5

**Table 3:** Data sources and the associated broad (numbers) and sub (lower case letters) Bartholomew natural history questions they aid in answering. Asterisks indicate data types/sources that need validated with additional information.

Data Type (Grey) and Source	N.H.? Source Can Answer
**Literature**	
Book—“*Amphibians and reptiles of*...”	1a; 2a–e; 3a–f; 4a–c; 5 a–f
Book—“*Field Guide to the Amphibians and reptiles*...”	1a; 2a–b,; 3d; 4b
Journal article—“*Behavioral ecology of*...”	1a; 2a–e; 3a–f; 4a–c; 5a–e.
Journal article—“*Community structure of*...”	1a,c; 2a–e; 3a–f; 4a–c; 5a–f.
Journal article—“*Ecology of*...”	1a; 2a–e; 3a–f; 4a–c; 5a–f
Journal article—“*Feeding ecology of*...”	1a; 2a–e; 3a–d,f; 4
Journal article—“*Diet of*…”	1a; 2a–e; 3a–d,f; 4
Journal article—“*Reproductive biology of*...”	1a; 2a–e; 3d–f; 4b; 5a–f
Journal article—Ecological modeling	1a,c; 2a–e; 3a–d,f; 4a–c; 5a–f
Journal article—Natural History note	1a; 2–e; 3a–d; 4b; 5a–e
Journal article—Species description/Taxonomic revision	1a–c; 2a–b,d–e; 3a,d,e; 4a–b; 5c,d–e.
**Virtual/Web-Based**	
Social Media—Facebook groups	1a–c*; 2a–e*; 3a–f*; 4a–c*; 5a–f*
Social Media—iNaturalist©	1a; 2a–b,e*; 3a–d*; 4b*
Website—Herpmapper©	1a; 2a–b,e*; 3a–d*; 4b*
Website—Natural history digital collection	2b–d*
Website/App—Taxon specific citizen science interfaces	1a–c*; 2a–e*; 3a–f*; 4a–c*; 5a–f*
Website/App—Weather stations/websites/Apps	2c–d*
**Field observations**	
In-situ field work by keeper	1a–c; 2a–b,d–e; 3a,d,e; 4a–b; 5c,d–e.

**Table 4:** Husbandry protocol for *Hydrodynastes gigas* using natural history information for its development. Justifications for each husbandry parameter are provided in the text. Parenthesis indicate source of information used for specific husbandry attribute. J = journal article; B = book/monograph; S = social media; Wb = website.

Husbandry Attribute	Determination
**1.What is needed for captive environment?**	
a. Enclosure type	PVC enclosure-ventilation needed. (J, S)
b. Enclosure dimensions (Length-Width-Height for adult)	8’ × 3’ × 2’ (J)
c. Substrate type	Mulch-wood pellet mix; sphagnum; leaf litter. (J, B, S)
d. Thermal needs—maintenance	31C high/ 22C low/ 25C ambient. (J, B, Wb)
e. Enclosure furniture type/amount	Minimum 2 hides, 1 cool; 1 humid; 1.0 mL, l–0.5 mW water tub. (J, S)
f. Light needs	5.0 UVB-LED bright light-light needed. (J, B)
g. Light cycle needs(L=light; D= Dark)	Outside of winter, 13 h L; 11 h D; winter 11 L; 13 D. (J, B, S)
h. Relative humidity	Moderate humidity needed year long (30–60%); Water basin
**2. What are the physiological needs?**	
a. Food items	Diverse—Staple diet laboratory rodents/ trout and frog legs. (J, S))
b. Feeding frequency	Twice weekly; one feeding of large prey (medium/large rat)/another feeding small prey item (Adult mouse/ S rat). (J)
c. Requirements for "normal" growth	Juveniles—two feedings/week - moderate to small prey items. (J)
**3. What is needed to encourage natural behaviors?**	
a. Space requirements	As much as possible - roaming needed. (J, B, S)
b. Hide availability	At least 2, 1 in each thermal zone. Substrate and cage furniture (Cork flats) also serve as hide. (J)
c. Thermal gradient	Ambient temperature needed to stay at/above 25C; cool and hot spots needed; water provides cool spot. (J, B, W)
d. Seasonal cycling—“Summer” cycle	February through October 13 h L/ 11 h D, 31 °C high/ 22 °C low/ 25 °C ambient food present weekly/biweekly (J, Wb)
e. Seasonal cycling—“Winter” cycle	Mid-November through January, 11 h D/13 h L, 25 °C high/ 15 °C low/ 21 °C ambient food present sparingly. (J, W)
**4. What are the welfare needs and concerns?**	
a. Space requirements	Neonates need privacy; As much as possible for juveniles’ adults. (J, S)
b. Privacy requirements	Multiple hides; deep substrates for burrowing. (J, Wb)
c. Methods for welfare assessment	Direct observation; Corticosterone testing of feces. (J)
d. Enrichment possibilities	Free roaming, prey encounter; scent presentation, conspecific sheds. (J)
**5. Captive breeding requirements**	
a. Seasonal cycling needed?	Yes—described above (J, Wb)
b. Incubation temperatures and duration?	28.1°C-75 days (J, S)
c. Feeding regime pre-mating	Cycle feeding—females heavy feeding 3 weeks prior to brumation. Two weekly feedings post ovulation. (J, S)
d. Care of neonates—ontogeny	Secure enclosures with ample ventilation & limited space; mulch wood pellet substrate; water bowl; used for 2–6 months

## Supplementary Materials

The following are available online at https://www.mdpi.com/2076-2615/10/11/2021/s1. Table S1: Example Table with husbandry questions for Evidence Based Herpetoculture Protocol Development.
