# Supplementary material for: Utilization of Natural History Information in Evidence based Herpetoculture: A Proposed Protocol and Case Study with Hydrodynastes gigas (False Water Cobra)
Source: Animals (Basel). 2020 Nov 3;10(11):2021. doi: 10.3390/ani10112021 (PMC7693199; doi:10.3390/ani10112021)
Supplement: Supplementary file 1 [file animals-10-02021-s001.pdf]

Husbandry Attribute
a. Enclosure type
b. Enclosure dimensions (L - W - H for adult)
b. Substrate type
c. Thermal needs - maintenance
d. Enclosure furniture type/amount
e. Light needs (UVB level; bulb type; etc.)
e. Light cycle needs
a. Food items
b. Feeding frequency
c. Requirements for "normal" growth
a. Space requirements
b. Hide availability
c. Thermal gradient
d. Seasonal cycling - "Summer" cycle
e. Seasonal cycling - "Winter" cycle
a. Space requirements
b. Privacy requirements
c. Methods for welfare assessment
d. Enrichment possibilities
a. Seasonal cycling needed?
b. Incubation temperatures and duration?
c. Feeding regime pre-mating
d. Care of neonates - ontogeny
Sources = J = journal article; B = book/monograph; S = social media; W = website.

Husbandry Question/Determination
1.What is needed for husbandry space?
2. What are the physiological needs?
3. What is needed to encourage natural behaviors?
4. What are the welfare needs and concerns?
5. Captive breeding requirements

Sources

Sources = J = journal article; B = book/monograph; S = social media; W = website.
